# Achieving consistency in measures of HIV‐1 viral suppression across countries: derivation of an adjustment based on international antiretroviral treatment cohort data

**DOI:** 10.1002/jia2.25776

**Published:** 2021-09-21

**Authors:** Leigh F. Johnson, Azar Kariminia, Adam Trickey, Constantin T. Yiannoutsos, Didier K. Ekouevi, Albert K. Minga, Ana Roberta Pati Pascom, Win Min Han, Lei Zhang, Keri N. Althoff, Peter F. Rebeiro, Gad Murenzi, Jonathan Ross, Nei‐Yuan Hsiao, Kimberly Marsh

**Affiliations:** ^1^ Centre for Infectious Disease Epidemiology and Research University of Cape Town Cape Town South Africa; ^2^ Kirby Institute University of New South Wales Sydney New South Wales Australia; ^3^ Population Health Sciences University of Bristol Bristol UK; ^4^ Fairbanks School of Public Health Indiana University Indianapolis Indiana USA; ^5^ Département de Santé Publique, Faculté des Sciences de la Santé Université de Lomé Lomé Togo; ^6^ Institut de Santé Publique, Epidémiologie et Développement (ISPED) Université de Bordeaux & Centre INSERM U1219 ‐ Bordeaux Population Health Bordeaux France; ^7^ Programme PAC‐CI Abidjan Côte d'Ivoire; ^8^ Department of Diseases of Chronic Condition and Sexually Transmitted Infections Ministry of Health Brasilia Brazil; ^9^ Johns Hopkins Bloomberg School of Public Health Baltimore Maryland USA; ^10^ Department of Medicine and Department of Biostatistics Vanderbilt University School of Medicine Nashville Tennessee USA; ^11^ Rwanda Military Hospital and Research for Development‐Rwanda Kigali Rwanda; ^12^ Division of General Internal Medicine Montefiore Medical Center/Albert Einstein College of Medicine Bronx New York USA; ^13^ Division of Virology University of Cape Town Cape Town South Africa; ^14^ National Health Laboratory Service Cape Town South Africa; ^15^ UNAIDS Geneva Switzerland

**Keywords:** antiretroviral therapy, HIV, viral load

## Abstract

**Introduction:**

The third of the Joint United Nations Programme on HIV/AIDS (UNAIDS) 90‐90‐90 targets is to achieve a 90% rate of viral suppression (HIV viral load <1000 HIV‐1 RNA copies/ml) in patients on antiretroviral treatment (ART) by 2020. However, some countries use different thresholds when reporting viral suppression, and there is thus a need for an adjustment to standardize estimates to the <1000 threshold. We aim to propose such an adjustment, to support consistent monitoring of progress towards the “third 90” target.

**Methods:**

We considered three possible distributions for viral loads in ART patients: Weibull, Pareto and reverse Weibull (imposing an upper limit but no lower limit on the log scale). The models were fitted to data on viral load distributions in ART patients in the International epidemiology Databases to Evaluate AIDS (IeDEA) collaboration (representing seven global regions) and the ART Cohort Collaboration (representing Europe), using separate random effects models for adults and children. The models were validated using data from the World Health Organization (WHO) HIV drug resistance report and the Brazilian national ART programme.

**Results:**

Models were calibrated using 921,157 adult and 37,431 paediatric viral load measurements, over 2010–2019. The Pareto and reverse Weibull models provided the best fits to the data, but for all models, the “shape” parameters for the viral load distributions differed significantly between regions. The Weibull model performed best in the validation against the WHO drug resistance survey data, while the Pareto model produced uncertainty ranges that were too narrow, relative to the validation data. Based on these analyses, we recommend using the reverse Weibull model. For example, if a country reports an 80% rate of viral suppression at <200 copies/ml, this model estimates the proportion virally suppressed at <1000 copies/ml is 88.3% (0.80^0.56^), with uncertainty range 85.5–90.6% (0.80^0.70^–0.80^0.44^).

**Conclusions:**

Estimates of viral suppression can change substantially depending on the threshold used in defining viral suppression. It is, therefore, important that viral suppression rates are standardized to the same threshold for the purpose of assessing progress towards UNAIDS targets. We have proposed a simple adjustment that allows this, and this has been incorporated into UNAIDS modelling software.

## 1 INTRODUCTION

Antiretroviral treatment (ART) is highly effective in preventing the replication of HIV, which is crucial to restoring the immune systems of people living with HIV [1], improving their long‐term survival [2] and reducing their risk of transmitting the virus to others [3]. Viral load tests, which measure levels of HIV‐1 RNA in the blood, are widely used to assess the effectiveness of ART. Because of the importance of viral suppression in reducing AIDS mortality and HIV transmission, the Joint United Nations Programme on HIV/AIDS (UNAIDS) has set the target of reaching 90% viral suppression in all ART patients by 2020 (the third of its “90‐90‐90” goals [4]) and increasing this proportion to 95% by 2025 [5].

Monitoring progress towards the third 90% target has been challenging. A major difficulty is that viral load data are typically missing for a large fraction of ART patients [6]. Another challenge is that countries use different assays and face varying delays in processing viral load specimens [7, 8]. These factors can affect viral load measurements at lower ranges of detection [9, 10], and can thus compromise comparability of viral suppression across settings. Finally, although the World Health Organization (WHO) and UNAIDS recommend reporting viral suppression at a threshold of less than 1000 HIV‐1 RNA copies/ml [11, 12], some countries have used lower thresholds in their reporting, which means that estimates of viral suppression are not consistently standardized.

The last of these challenges has been a particular concern of the UNAIDS Reference Group on Estimates, Models and Projections, which oversees the UNAIDS estimation process. Prior to 2019, the UNAIDS estimates of progress towards the third 90% target did not adjust for differences in the threshold countries used for defining viral suppression [6]. There was a desire for a simple formula that could be used to standardize these estimates of viral suppression to the “less than 1000” threshold. It was also anticipated that such a formula might become important if the WHO recommendations were to switch to a different reporting threshold in the future, which would necessitate adjustments to historic estimates for the purpose of assessing trends in viral suppression.

This study aims to compare alternative models for standardizing estimates of viral suppression to the same threshold. For each model, we estimate the model parameters using data from two of the largest global ART collaborations: the International epidemiology Databases to Evaluate AIDS (IeDEA) collaboration and the ART Cohort Collaboration (ART‐CC). Based on comparisons of likelihood statistics and validations using data from smaller studies, we assess which models are best, and describe the adjustment approach that UNAIDS has adopted.

## 2 METHODS

### 2.1 Adjustment approaches

We consider three possible models of viral load distributions, limiting our focus to mathematical forms that allow a simple adjustment of the proportion virally suppressed. The first model is a Weibull model. If the viral loads in treated patients (on a log_10_ scale) are Weibull‐distributed with shape parameter *ϕ* and scale parameter *λ*, then the probability of the viral load being below threshold *t*_1_, *F*(*t*_1_), is shown in Table 1. If *ϕ* is considered a known parameter, *λ* can be calculated from *ϕ* and *F*(*t*_1_), and this is used to calculate the adjusted estimate of viral suppression at the alternative threshold of *t*_2_ (Table 1).

**Table 1:** Models of viral load distributions in ART patients

	Weibull	Reverse Weibull	Pareto
Probability of viral load below threshold *t*_1_, *F*(*t*_1_)	$1-\exp(-\lambda \;\log_{10}{\left(t_{1}\right)}^{\phi })$	$\exp(-\lambda {\left(6-\log_{10}\left(t_{1}\right)\right)}^{\phi })$	$1-{\left(\frac{m}{\log_{10}\left(t_{1}\right)}\right)}^{\alpha }$
Shape parameter	*ϕ*	*ϕ*	*α*
Scale parameter, if *F*(*t*_1_) and shape parameter are known	$\lambda =\frac{-\ln(1-F(t_{1}))}{\log_{10}{\left(t_{1}\right)}^{\phi }}$	$\lambda =\frac{-\ln(F(t_{1}))}{{\left(6-\log_{10}\left(t_{1}\right)\right)}^{\phi }}$	$m=\log_{10}\left(t_{1}\right){\left(1-F(t_{1})\right)}^{\frac{1}{\alpha }}$
Probability of viral load below threshold *t*_2_, *F(t_2_)*, if *F*(*t*_1_) and shape parameter are known	$1-\left(1-F(t_{1})\right)\begin{array}{c} {}^{{\left(\frac{\log_{10}\left(t_{2}\right)}{\log_{10}\left(t_{1}\right)}\right)}^{\phi }} \end{array}$	$F\left(t_{1}\right)\begin{array}{c} {}^{{\left(\frac{6-\log_{10}\left(t_{2}\right)}{6-\log_{10}\left(t_{1}\right)}\right)}^{\phi }} \end{array}$	$1-\left(1-F(t_{1})\right){\left(\frac{\log_{10}\left(t_{1}\right)}{\log_{10}\left(t_{2}\right)}\right)}^{\alpha }$
Lower limit	1	0	∼5
Upper limit	None	1,000,000	None

Note: For all three models, the shape parameter controls the variance of the distribution of viral loads (a higher shape parameter implies a lower ratio of the standard deviation to the mean). The scale parameter (*λ* for the Weibull and reverse Weibull models, *m* for the Pareto model) determines the mean of the distribution (a higher scale parameter implies a higher mean viral load for the Weibull and Pareto models, but a lower mean viral load for the reverse Weibull model). For the Pareto distribution, the lower limit is 10*^m^*, which in most cohorts is estimated to be around 5 copies/ml.

**Figure 1:**
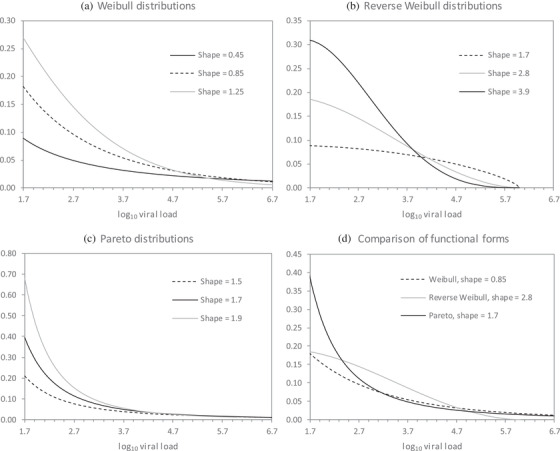
Probability density functions for viral loads in ART patients, under different statistical models. Probability density functions with different shape parameters are presented for illustrative purposes. In all cases, the *λ* or *m* parameter has been set so that the cumulative probability of a viral load less than 1000 copies/ml is the same (0.85). The probability densities are truncated at 50 copies/ml (1.7 on the log_10_ scale), as different models impose different lower limits, and lower limits below 50 copies/ml have not been used for reporting purposes.

Figure 1a illustrates the probability density functions for Weibull distributions with different shape parameters (in all cases, the *λ* parameter has been calculated to yield the same probability of viral suppression at <1000 copies/ml). A limitation of this model is that it does not reflect realistic upper limits on the viral load, which seldom exceeds 1,000,000 copies/ml (6 on the log_10_ scale) [9, 13, 14], while the density function has a lower limit of 1 copy/ml (0 on the log_10_ scale).

In an attempt to impose more natural limits on the viral load, we consider an alternative reverse Weibull model. If *X* is the difference between a patient's viral load (on the log_10_ scale) and a notional upper limit of 6 on the log_10_ scale, and *X* is Weibull‐distributed with shape parameter *ϕ* and scale parameter *λ*, the equations for this model are given in Table 1.

Figure 1b illustrates the probability density functions for reverse Weibull distributions with different shape parameters.

The final model is a Pareto model. Table 1 shows the equations for a Pareto distribution with shape parameter *α* and scale parameter *m*. Figure 1c illustrates the probability density functions of Pareto distributions with different shape parameters. As with the Weibull distribution, this distribution can yield implausibly high viral loads in treated patients. In addition, the probability density function has a lower limit of 10*^m^*, which for most plausible values of *m* yields a lower limit of around 5 copies/ml. Figure 1d compares the Weibull, reverse Weibull and Pareto models.

### 2.2 Data sources for model calibration and validation

Models were calibrated using data from the IeDEA collaboration and ART‐CC, from 2010 onwards. IeDEA is a large collaboration of ART programmes, divided into seven regions: Asia‐Pacific; Caribbean, central America and South America (“CCASAnet”); central Africa; East Africa; North America; southern Africa; and West Africa [15]. ART‐CC is a collaboration of ART programmes in Europe [16]. All cohorts participating in IeDEA and ART‐CC received local institutional approval to share anonymized data. For each year from 2010, participating cohorts contributed data on the numbers of ART patients who received a viral load (with a maximum of one viral load per patient per year), and the number of viral load measurements in the <50, 50–199, 200–399, 400–999 and ≥1000 categories, separately for adults and children. Cohorts that used viral load assays with lower detection limits above 50 copies/ml provided viral load counts that were categorized as <400, 400–999 or ≥1000. Data from cohorts that conducted targeted viral load monitoring were excluded; these were mostly in sub‐Saharan Africa [15].

The best‐fitting models were validated by comparing the model predictions against data from two sources that could be accessed in time for the 2021 UNAIDS estimation process. The first was the WHO HIV Drug Resistance Report, which collected data on viral suppression at different thresholds in five countries, at 12 and 48 months after ART initiation, in 2016 [17]. The second was the Brazilian national ART programme, with data from 2014 to 2019 [18].

### 2.3 Statistical analysis

A multinomial likelihood was specified to represent the degree of consistency between the model estimates of the numbers of patients in each viral load compartment and the observed numbers. For each of the eight regions, we separately fitted the Weibull, reverse Weibull and Pareto models to the IeDEA/ART‐CC data, to find the shape parameter that maximized the likelihood, allowing for random effects (in the *λ* or *m* parameters). Subjects were grouped by ART programme and calendar year, and differences between groups were modelled as random effects.

Shape parameters estimated for each of the eight regions were averaged in a pooled analysis, using the “metan” meta‐analysis command in STATA [19], with region‐specific random effects. Prediction intervals were calculated to reflect both the variance in random effects across regions and the uncertainty around the true mean. The models were validated by substituting the observed proportions of patients virally suppressed (*F*(*t*_1_)) at lower thresholds (*t*_1_) into each equation for *F*(*t*_2_) (Table 1), using the respective mean shape parameters, and setting threshold *t*_2_ = 1000. Uncertainty ranges were calculated by similarly substituting the lower and upper bounds of the 95% prediction intervals around the shape parameters into equations for *F*(*t*_2_). The resulting adjusted estimates of viral suppression (at the threshold of <1000 copies/ml) were compared against the observed proportions, and the extent of the divergence between the predictions and the observed proportions was quantified using the root‐mean‐square error (RMSE). All statistical analyses were conducted using STATA 15.1 (StataCorp, College Station, TX, USA) and Microsoft Excel (Microsoft Corporation, Redmond, WA, USA).

## 3 RESULTS

Models were calibrated using 921,157 adult viral load measurements and 37,431 paediatric viral load measurements (Table 2).

**Table 2:** Data summary

	Period	Programme–year combinations^a^	Total viral loadmeasurements	Viral suppression (<1000 copies/ml)
Adult data				
Asia‐Pacific	2010–2019	13	6860	97.0%
CCASAnet	2010–2019	10	32,958	90.4%
Central Africa	2016–2019	26	3600	92.6%
East Africa	2013–2019	30	85,258	91.2%
North America	2010–2018	117	35,168	89.3%
Southern Africa	2010–2019	54	499,112	90.1%
West Africa	2010–2018	25	7446	91.7%
Europe	2010–2019	74	250,755	93.9%
Total		349	921,157	91.6%
Paediatric data				
Asia‐Pacific	2010–2019	10	4811	86.6%
CCASAnet	2011–2018	8	520	76.9%
Central Africa	2017–2019	3	205	83.9%
East Africa	2014–2019	17	7921	75.6%
Southern Africa	2010–2019	60	20,827	75.5%
West Africa	2011–2018	36	3147	66.0%
Total		134	37,431	76.2%

^a^ Calculated by summing the number of calendar years that each programme contributes data; a separate random effect is fitted for each programme–year combination.

Abbreviation: CCASAnet, Caribbean, Central America and South America network.

Table 3 shows the best‐fitting shape parameters for each model, together with log likelihood statistics (the best‐fitting models are indicated in bold). In most regions, the Pareto model provided the best fit to the adult viral load data, although the reverse Weibull model provided the best fit to the adult data in eastern and southern Africa. The shape parameters estimated by the Pareto and reverse Weibull models were highest in the regions with the highest rates of viral suppression (Asia‐Pacific and Europe), while the shape parameters estimated by the Weibull model were not correlated with levels of viral suppression. Shape parameters differed between regions, for all three models (*p* < 0.001). In the pooled analysis, the average of the best‐fitting parameters was 0.85 (95% prediction interval: 0.43 to 1.26) for the Weibull model, 2.81 (1.70–3.92) for the reverse Weibull model and 1.73 (1.20–2.26) for the Pareto model. Figure 1 illustrates the interpretation of these shape parameters.

**Table 3:** Estimates of model parameters

	Weibull model		Reverse Weibull model		Pareto model	
	Log L	Shape (*ϕ*)	Log L	Shape (*ϕ*)	Log L	Shape (*α*)
Adult data						
Asia‐Pacific	–2823	0.74 (0.68–0.80)	–2842	2.98 (2.91–3.06)	**–2797**	2.12 (1.92–2.33)
CCASAnet	–26,621	0.84 (0.82–0.86)	–26,408	2.86 (2.78–2.94)	**–26,227**	1.52 (1.48–1.57)
Central Africa	–2510	0.80 (0.73–0.87)	–2525	3.01 (2.72–3.29)	**–2499**	1.68 (1.51–1.84)
East Africa	–82,596	1.24 (1.23–1.26)	**–82,314**	3.05 (3.05–3.06)	–82,454	1.95 (1.92–1.98)
North America	–19,173	0.76 (0.73–0.79)	–19,191	2.31 (2.20–2.41)	**–19,095**	1.48 (1.41–1.54)
Southern Africa	–144,959	0.74 (0.73–0.76)	**–144,339**	2.07 (2.03–2.11)	–144,432	1.60 (1.56–1.63)
West Africa	–4045	0.69 (0.63–0.74)	–4051	2.52 (2.30–2.74)	**–4001**	1.49 (1.35–1.62)
Europe	–151,923	0.96 (0.96–0.97)	–152,331	3.70 (3.66–3.74)	**–150,052**	2.05 (2.02–2.07)
Average^a^		0.85 (0.43–1.26)		2.81 (1.70–3.92)		1.73 (1.20–2.26)
Paediatric data						
Asia‐Pacific	–2580	0.57 (0.49–0.65)	–2585	1.48 (1.26–1.71)	**–2574**	1.04 (0.88–1.20)
CCASAnet	–527	0.64 (0.50–0.78)	**–526**	1.49 (1.15–1.84)	–527	0.77 (0.58–0.95)
Central Africa	–187	0.72 (0.47–0.97)	–189	2.00 (1.27–2.74)	**–187**	1.08 (0.67–1.49)
East Africa	–6893	1.04 (0.97–1.11)	**–6860**	2.06 (1.92–2.21)	–6883	1.31 (1.22–1.41)
Southern Africa	–18,109	1.22 (1.18–1.27)	**–17,965**	2.32 (2.22–2.41)	–18,000	1.29 (1.24–1.35)
West Africa	–3379	0.89 (0.81–0.96)	–3388	1.65 (1.51–1.80)	**–3373**	0.79 (0.72–0.87)
Average^a^		0.85 (0.26–1.45)		1.84 (1.03–2.64)		1.05 (0.46–1.64)

^a^ Average calculated by meta‐analysis. Log L = log likelihood (values in bold indicate the model that gives the highest log likelihood). 95% confidence intervals around shape parameters are shown in parentheses.

Abbreviation: CCASAnet, Caribbean, Central America and South America network.

The reverse Weibull and Pareto models also provided the best fits to the paediatric viral suppression data. In the Weibull and reverse Weibull models, shape parameters were consistently higher in the African regions than in the non‐African regions. However, shape parameters were not correlated with levels of paediatric viral suppression, for any of the three models. In the pooled analysis, the average of the best‐fitting parameters was 0.85 (0.26–1.45) for the Weibull model, 1.84 (1.03–2.64) for the reverse Weibull model and 1.05 (0.46–1.64) for the Pareto model.

**Figure 2:**
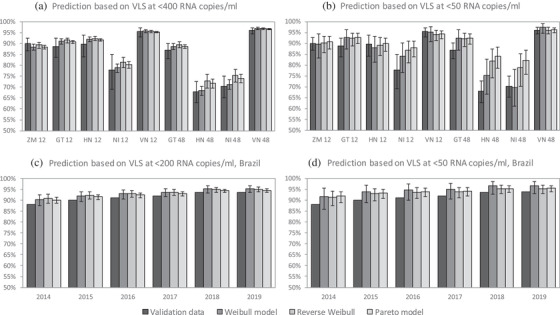
Validation of the model predictions of viral suppression (at <1000 copies/ml) against data from the WHO HIV Drug Resistance Report (panels a, b) and Brazilian programme data (panels c, d). In panels a and b, results are presented by country code (GT = Guatemala, HN = Honduras, NI = Nicaragua, VN = Vietnam, ZM = Zambia) and ART duration (in months). Confidence intervals around the validation data are not shown in panels c and d, as these estimates are based on large patient numbers and standard error estimates are <0.1%. Abbreviation: VLS, viral load suppression.

The calibrated adult models were applied to WHO data on the proportion of adults on ART suppressed at <400 copies/ml, using the equations for *F*(*t*_2_) in Table 1 to predict the proportions virally suppressed at <1000 copies/ml. These predictions were compared to the observed proportions suppressed at <1000 copies/ml in the WHO survey data (Figure 2a). The Weibull model provided the closest correspondence (RMSE 1.5% compared to 3.1% for the reverse Weibull model and 2.4% for the Pareto model), although none of the model predictions differed significantly from the survey data. The models were also applied to the data on the proportions suppressed at <50 copies/ml, and the resulting predictions of suppression at <1000 copies/ml were compared against the survey data (Figure 2b). The Weibull model predictions were again closest to the data (RMSE 4.0% compared to 6.6% for the reverse Weibull model and 7.9% for the Pareto model), and the Pareto model predictions differed significantly from the data points in a number of cases.

The adult models were also applied to the Brazilian programme data on the proportions suppressed at <200 copies/ml, to produce predictions of the proportions virally suppressed at <1000 copies/ml. These predictions were consistently higher than the observed proportions suppressed, for all three models (Figure 2c). Although the Pareto model point estimates were closer to the data than the other models (RMSE 1.3% compared to 1.8% for the Weibull model and 2.0% for the reverse Weibull model), the uncertainty ranges around the Pareto model predictions were narrower than for the other two models. This meant that the lower limits of the Pareto uncertainty ranges were no closer to the validation data than those of the other models. Similarly, the models were applied to the programme data on the proportions suppressed at <50 copies/ml, and resulting predictions of suppression at <1000 copies/ml were compared against the programme data. Again, all three models over‐estimated the actual proportions virally suppressed, although the uncertainty ranges around the Weibull and reverse Weibull predictions mostly included the data points (Figure 2d). The reverse Weibull model was closest to the data (RMSE 2.4% compared to 3.2% for the Weibull model and 2.6% for the Pareto model). Model predictions of increases in viral suppression, as a result of moving to the <1000 threshold, are compared against actual increases in Figure S1.

**Figure 3:**
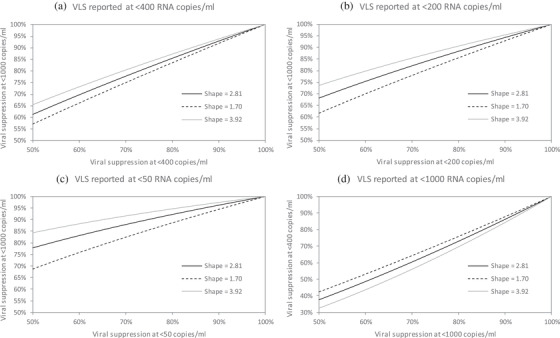
Reverse Weibull adjustments, with uncertainty ranges. In each panel, the solid black line represents the point estimate for the adjusted viral suppression, based on a reported rate of viral suppression at a threshold specified on the *x* axis. The upper and lower lines represent the uncertainty ranges around the point estimates, calculated from the 95% prediction intervals around the shape parameter. In panel a, the adjusted rates of viral suppression are calculated using the formulas *F*(*t*_2_) = *F*(*t*_1_)^0.70^, *F*(*t*_2_) = *F*(*t*_1_)^0.81^ and *F*(*t*_2_) = *F*(*t*_1_)^0.61^, for shape parameters 2.81, 1.70 and 3.92, respectively, calculated by substituting the relevant shape parameters into the equation for *F*(*t*_2_). In panel b, the adjusted rates of viral suppression are calculated using the formulas *F*(*t*_2_) = *F*(*t*_1_)^0.56^, *F*(*t*_2_) = *F*(*t*_1_)^0.70^ and *F*(*t*_2_) = *F*(*t*_1_)^0.44^, for shape parameters 2.81, 1.70 and 3.92, respectively, and in panel c, the adjusted rates are calculated using the formulas *F*(*t*_2_) = *F*(*t*_1_)^0.36^, *F*(*t*_2_) = *F*(*t*_1_)^0.54^ and *F*(*t*_2_) = *F*(*t*_1_)^0.24^. Panel d represents an alternative scenario in which viral suppression is reported at a threshold of <1000 copies/ml, but we wish to adjust the reported rate to obtain an estimate of viral suppression at <400 copies/ml; here, the formulas are *F*(*t*_2_) = *F*(*t*_1_)^1.42^, *F*(*t*_2_) = *F*(*t*_1_)^1.24^ and *F*(*t*_2_) = *F*(*t*_1_)^1.63^, respectively. Abbreviation: VLS, viral load suppression.

Because of the relatively poor log likelihood statistics for the Weibull model (Table 3) and because the uncertainty ranges around the Pareto model predictions generally appeared too narrow relative to the validation data (Figure 2), the UNAIDS Reference Group selected the reverse Weibull model as the default model for adjusting adult viral load suppression estimates. Figure 3 illustrates the effect of applying these reverse Weibull adjustments at different viral load levels, using the shape parameter (and 95% prediction interval limits) from Table 3. For example, if a programme reports that 80% of adults have viral loads <200 copies/ml, and we wish to estimate the fraction who have viral loads <1000 copies/ml, substituting *t*_1_ = 200, *t*_2_ = 1000 and *ϕ* = 2.81 into the equation for *F*(*t*_2_) yields an adjusted estimate of 88.3%. Replacing the shape parameter with 1.70 and 3.92 yields lower and upper limits of 85.5% and 90.6%, respectively. The same adjustment can be used in situations where the reported threshold is <1000 copies/ml and we wish to standardize to a lower threshold, for example <400 copies/ml (Figure 3d).

## 4 DISCUSSION

In this analysis, we compared three approaches to estimating viral suppression standardized to a threshold of <1000 copies/ml, finding that a reverse Weibull model performed best overall. Our analysis demonstrates that estimates of viral suppression can change substantially depending on the threshold used to define viral suppression. In the example presented, a country that reports that 80% of adults on ART are virally suppressed at a threshold of 200 copies/ml might appear to be performing poorly relative to the UNAIDS 90% target. However, after adjustment, the fraction virally suppressed at the standard threshold of <1000 copies/ml increases to 88.3%, which appears much more favourable. It is, therefore, important that rates of viral suppression are standardized to the <1000 threshold for the purpose of assessing progress towards the UNAIDS targets, as well as when comparing rates of viral suppression across countries. This paper proposes an adjustment approach that is simple and statistically rigorous, and that has been calibrated using data from the two largest ART collaborations globally. The adjustment approach also includes an uncertainty range, to reflect variability in the “shape” of viral load distributions across settings. The recommended adjustment has been incorporated into the Spectrum model, which is supported by UNAIDS and widely used in producing HIV estimates nationally and globally [20].

An important finding is that the shape of the viral load distribution in people on ART can differ substantially across settings. Previous studies have focused on variations in average levels of viral suppression between regions and groups of ART patients [15, 21, 22], but Figure 1 demonstrates that there can be substantial differences in viral load distributions even when the fraction virally suppressed is the same. There have been few attempts to understand what factors drive these differences in shape. One factor may be the test turnaround time (the time from when the specimen is collected to when the test is completed). South African data suggest that patients with low levels of viraemia might be incorrectly classified as “undetectable” when there are delays in processing viral load specimens [10]. This could lead to the variance of the viral load distribution being exaggerated, which would imply a smaller shape parameter (assuming all other things being the same). Similar concerns about inconsistent performance of assays at lower limits of detection have been noted in other studies [9].

Another factor accounting for differences in the shape of the viral load distribution may be differences in the prevalence of drug resistance, as patients with drug‐resistant virus are more likely to have unsuppressed viraemia levels in the intermediate range [23]. Levels of drug resistance are likely to be highest at longer ART durations [24] and when drugs have a lower genetic barrier to resistance. ART programmes that have longer average ART durations and that have not yet transitioned to dolutegravir may thus have higher levels of drug resistance [25] and hence, a differently shaped distribution of viral loads. Further work is required to assess whether different adjustments may be appropriate depending on factors such as the level of drug resistance and the extent of dolutegravir rollout.

Although the default adjustments recommended here are based on adult data, different adjustments may be appropriate in children. Levels of viral suppression are lower in children than in adults (Table 2), and the shape parameters for the reverse Weibull and Pareto models are correspondingly lower in children (Table 3). This could reflect greater heterogeneity in viral suppression in children, with levels of viral suppression being particularly low in young children (<5 years) and adolescents [15, 21], and with children taking longer to achieve viral suppression after ART initiation than adults [26]. Global reporting has previously focused on aggregated indicators of viral suppression, but new guidelines recommend monitoring progress towards the 95% target separately for adults and children [5]. These results could be important in future standardization of paediatric viral suppression estimates.

Although the UNAIDS Reference Group on Estimates, Modelling and Projections has recommended the reverse Weibull model as the default for standardizing viral load measurements to the same threshold, one could make strong arguments for using the Weibull or Pareto models instead. The Pareto model provides a better fit to the adult IeDEA and ART‐CC data than the reverse Weibull model (Table 3), and the Weibull model was more consistent with the WHO HIV drug resistance report data in the validation (Figure 2a, b). However, the reverse Weibull model is more theoretically appealing than the other two models, as it does not lead to implausible upper and lower bounds on the viral load distribution. These implausible bounds are not a major concern if we are only considering the commonly used thresholds for reporting viral suppression, but could be important in modelling viral load distributions in ART patients more generally, for example when assessing the extent to which ART reduces HIV transmission risks [27, 28].

Another advantage of the reverse Weibull model is that the adjustment is mathematically simpler than the other adjustment models (the equations for the adjustments are included in Figure 3 footnotes). We have limited our focus to distributional forms that would lead to simple adjustments. Although there are many other statistical distributions that could be used to describe viral load distributions in ART patients (that is log‐normal, gamma and generalized gamma), these would not lead to simple adjustments.

A potential criticism of the reverse Weibull and Pareto models is that the estimated shape parameters are correlated with the levels of viral suppression, in contrast to the Weibull shape parameters, which are not correlated with levels of viral suppression. This is a potential concern, as the IeDEA and ART‐CC collaborations mostly represent cohorts in which high levels of viral suppression have been achieved (92% as compared to a global average of 86% in 2018 [6]). The reverse Weibull and Pareto shape parameters that have been estimated from the IeDEA and ART‐CC data might, therefore, be less applicable in settings where viral suppression is low. In the validation against the WHO HIV drug resistance data, the reverse Weibull and Pareto models performed relatively poorly in settings with low viral suppression (Nicaragua and Honduras), while the Weibull model was more consistent with the validation data in these settings (Figure 2a, b).

None of the models performed well in predicting the levels of viral suppression in Brazil, although the observed levels of viral suppression were at least within the model uncertainty ranges in most cases for the Weibull and reverse Weibull models. A general concern is that the validation relies heavily on data from South and central America, where levels of drug resistance are relatively high [24, 25, 29]. These high levels of drug resistance might explain why our model appears to “over‐adjust” in many countries in the region, given the previously noted effects of drug resistance. It will be important to do further validations in future, using additional data from other regions. A further limitation is that this analysis is based on aggregated data, and because it was not possible to obtain individual‐level data, we could not perform sub‐analyses to assess which factors might account for variations in shape parameters across regions. A hypothesized factor that might explain this variation is the type of viral load assay used [13], but only one IeDEA region (Asia‐Pacific) was able to provide detailed information on this. Disaggregation of results by sex was possible in southern Africa, but no substantial sex differences in the shape parameter were found (Table S1).

The need for further validation and sub‐analysis reflects a broader need for better viral load data. Where possible, countries should use actual data on the proportions virally suppressed at the recommended reporting threshold, rather than relying on the statistical adjustment proposed in this paper, and our endorsement of this adjustment does not remove the need to collect these data.

## 5 CONCLUSIONS

As UNAIDS moves towards increasingly ambitious targets for achieving viral suppression, and as countries get closer to achieving these targets, it becomes increasingly important to ensure that countries use the same measure of viral suppression. This study proposes a simple adjustment that can be used not only to standardize viral load measures to the current reporting threshold of <1000 copies/ml, but also to standardize to alternative thresholds. The latter is likely to be important if WHO recommends switching to using a reporting threshold of <400 copies/ml in the future.

## COMPETING INTERESTS

The authors declare that no competing interests exist.

## AUTHORS' CONTRIBUTIONS

LFJ and KM conceived the study. AK, AT, CTY, DKE, AKM, ARP, WMH, LZ, KNA, PFR, GM and JR contributed to data collection. LFJ, NH and KM contributed to data analysis. LFJ with input from all authors drafted the manuscript. All authors have read and approved the final manuscript.

## FUNDING

ART‐CC: The ART‐CC isfunded by the US National Institute on Alcohol Abuse and Alcoholism (U01‐AA026209). Sources of funding of individual cohorts include the ANRS (France REcherche Nord&Sud Sida‐hiv Hépatites), the Institut National de la Santé et de la Recherche Médicale (INSERM), the French, Italian, and Spanish Ministries of Health, the Preben and Anne Simonsens Foundation, the Ministry of Science and Innovation and the Spanish Network for AIDS Research [Spanish Network of Excellence on HIV (RD12/0017/0018, RD16CIII/0002/0006)], and unrestricted grants from Abbott, Gilead, Tibotec‐Upjohn, ViiV Healthcare, MSD, GlaxoSmithKline, Pfizer, Bristol‐Myers Squibb, Roche and Boehringer Ingelheim.

Asia‐Pacific: The TREAT Asia HIV Observational Database is an initiative of TREAT Asia, a program of amfAR, The Foundation for AIDS Research, with support from the U.S. National Institutes of Health's National Institute of Allergy and Infectious Diseases (NIAID), the Eunice Kennedy Shriver National Institute of Child Health and Human Development (NICHD), the National Cancer Institute (NCI), the National Institute of Mental Health (NIMH), and the National Institute on Drug Abuse (NIDA), the National Heart, Lung, and Blood Institute (NHLBI), the National Institute on Alcohol Abuse and Alcoholism (NIAAA), the National Institute of Diabetes and Digestive and Kidney Diseases (NIDDK), and the Fogarty International Center (FIC), as part of the International Epidemiology Databases to Evaluate AIDS (IeDEA; U01AI069907). The Kirby Institute is funded by the Australian Government Department of Health and Ageing, and is affiliated with the Faculty of Medicine, UNSW Sydney.

Caribbean, Central America and South America: This work was supported by the NIH‐funded Caribbean, Central and South America network for HIV epidemiology (CCASAnet), a member cohort of leDEA (U01AI069923). This award is funded by the following institutes: NIAID, NICHD, NCI, NIMH, NIDA, NHLBI, NIAAA, NIDDK, FIC and the National Library of Medicine (NLM). Peter Rebeiro was supported by NIH Award Number K01AI131895 (“The HIV Care Continuum and Health Policy: Changes Through Context and Geography”).

Central Africa: Research reported in this publication was supported by NIAID of the National Institutes of Health under Award Number U01AI096299 (PI: Anastos, Nash and Yotebieng). This award is funded by the following institutes: NIAID, NICHD, NCI, NIMH, NIDA, NHLBI, NIAAA, NIDDK, FIC and NLM.

East Africa: Research reported in this publication was supported by NIAID, NICHD, NIDA, NCI and NIMH, in accordance with the regulatory requirements of the US NIH under Award Number U01AI069911 East Africa IeDEA Consortium.

North America: This work was supported by US NIH grants U01AI069918, F31AI124794, F31DA037788, G12MD007583, K01AI093197, K01AI131895, K23EY013707, K24AI065298, K24AI118591, K24DA000432, KL2TR000421, M01RR000052, N01CP01004, N02CP055504, N02CP91027, P30AI027757, P30AI027763, P30AI027767, P30AI036219, P30AI050410, P30AI094189, P30AI110527, P30MH62246, R01AA016893, R01CA165937, R01DA011602, R01DA012568, R01 AG053100, R24AI067039, U01AA013566, U01AA020790, U01AI031834, U01AI034989, U01AI034993, U01AI034994, U01AI035004, U01AI035039, U01AI035040, U01AI035041, U01AI035042, U01AI037613, U01AI037984, U01AI038855, U01AI038858, U01AI042590, U01AI068634, U01AI068636, U01AI069432, U01AI069434, U01AI103390, U01AI103397, U01AI103401, U01AI103408, U01DA03629, U01DA036935, U01HD032632, U10EY008057, U10EY008052, U10EY008067, U24AA020794, U54MD007587, UL1RR024131, UL1TR000004, UL1TR000083, UL1TR000454, UM1AI035043, Z01CP010214 and Z01CP010176; contracts CDC‐200‐2006‐18797 and CDC‐200‐2015‐63931 from the Centers for Disease Control and Prevention, USA; contract 90047713 from the Agency for Healthcare Research and Quality, USA; contract 90051652 from the Health Resources and Services Administration, USA; grants CBR‐86906, CBR‐94036, HCP‐97105 and TGF‐96118 from the Canadian Institutes of Health Research, Canada; Ontario Ministry of Health and Long Term Care; and the Government of Alberta, Canada. Additional support was provided by NCI, NIMH and NIDA.

Southern Africa: Research reported in this publication was supported by NIAID of the US NIH under Award Number U01AI069924.

West Africa: Research reported in this publication was supported by the US NIH (NIAID, NICHD, NCI, NHLBI, NIDDK, NIAAA, FIC and NIMH) under Award Number U01AI069919 (PI: Dabis).

IeDEA informatics resources are supported by the Harmonist project, R24AI124872.

## DISCLAIMER

This work is solely the responsibility of the authors and does not necessarily represent the official views of any of the institutions mentioned above.

## Supporting information

**Table S1**. Estimates of model parameters in Southern African adults
**Table S2**. Estimates of model parameters in European adults
**Figure S1**. Percentage point changes in the proportion of adults
