# Supplementary material for: Achieving consistency in measures of HIV‐1 viral suppression across countries: derivation of an adjustment based on international antiretroviral treatment cohort data
Source: J Int AIDS Soc. 2021 Sep 21;24(Suppl 5):e25776. doi: 10.1002/jia2.25776 (PMC8454679; doi:10.1002/jia2.25776)
Supplement: Supplementary file 1 — Table S1. Estimates of model parameters in Southern African adults Table S2. Estimates of model parameters in European adults Figure S1. Percentage point changes in the proportion of adults [file JIA2-24-e25776-s001.docx]

Supplementary materials

These supplementary materials provide additional results to complement the main results of the paper ‘Achieving consistency in measures of HIV-1 viral suppression across countries: derivation of an adjustment based on international antiretroviral treatment cohort data’ in *Journal of the International AIDS Society*.

Although the data requested from IeDEA did not include stratification by sex, it was possible to obtain adult viral load data stratified by sex for the Southern African region, which accounted for 54% of all the adult data in the main analysis. Table S1 below shows the sex-stratified results for adults in IeDEA Southern Africa, in the same format as Table 3 of the main text. It is clear that for each model the shape parameters are very similar for males and females. Although the difference between the male and female shape parameters is statistically significant in the case of the Pareto model, this is nevertheless a less than 10% difference in shape parameters, which is small relative to the variation in shape parameters across regions (see Table 3 of the main text). Differences in the sex ratio of ART patients across regions are thus unlikely to explain the differences in shape parameters across regions. The table also shows that the reverse Weibull model is still the best-fitting model, both for males and females, and thus the choice of model does not appear to be influenced by sex disaggregation.

Table S1: Estimates of model parameters in Southern African adults, stratified by sex

|  | Weibull model | | Reverse Weibull model | | Pareto model | |
| --- | --- | --- | --- | --- | --- | --- |
|  | Log L | Shape (*ϕ*) | Log L | Shape (*ϕ*) | Log L | Shape (*m*) |
| Males | -45303 | 0.78 (0.76-0.81) | **-45113** | 2.01 (1.94-2.08) | -45126 | 1.51 (1.46-1.56) |
| Females | -94158 | 0.75 (0.74-0.77) | **-93798** | 2.12 (2.07-2.17) | -93880 | 1.65 (1.61-1.69) |

Log L = log likelihood (values in bold indicate the model that gives the highest log likelihood). 95% confidence intervals around shape parameters are shown in parentheses.

The IeDEA data we requested also do not include disaggregation by drug or drug class. We were however able to approximate the effect of dolutegravir rollout using the data from ART-CC (Europe), which supplied information on the proportion of treatment initiators whose first-line regimen contained dolutegravir (which we take to be a proxy for the proportion of all ART patients on dolutegravir). For the purpose of analysing these data, we have grouped each cohort and each year of viral load data into one of three categories: less than 10% of new ART initiators starting dolutegravir, 10-39% of new ART initiators starting dolutegravir and 40% or more of new ART initiators starting dolutegravir. The different statistical models were fitted separately for each of the three groups, but no consistent trend emerged (Table S2). In all three models the shape parameter increased when comparing the 10-39% category to the <10% category (implying a reduction in the coefficient of variation), but the shape parameter reduced when comparing the ≥40% category to the 10-39% category. Although there is no clear ‘dose-response’ relationship, this does not exclude the possibility of a dolutegravir effect, since we have relatively few programme-year combinations in which there was high dolutegravir coverage, and other factors may be confounding the relationship between dolutegravir coverage and the shape parameter.

Table S2: Estimates of model parameters in European adults, stratified by level of dolutegravir rollout

| DTG | n^*^ | Weibull model | | Reverse Weibull model | | Pareto model | |
| --- | --- | --- | --- | --- | --- | --- | --- |
| rollout |  | Log L | Shape (*ϕ*) | Log L | Shape (*ϕ*) | Log L | Shape (*m*) |
| <10% | 34 | **-79719** | 0.96 (0.95-0.98) | -79918 | 3.32 (3.27-3.37) | -78910 | 1.81 (1.78-1.84) |
| 10-39% | 19 | -41188 | 0.98 (0.96-1.00) | -41199 | 4.17 (4.08-4.27) | **-40485** | 2.32 (2.27-2.37) |
| ≥40% | 12 | -23614 | 0.86 (0.84-0.89) | -23647 | 3.73 (3.62-3.85) | **-23274** | 2.10 (2.03-2.16) |

Log L = log likelihood (values in bold indicate the model that gives the highest log likelihood). 95% confidence intervals around shape parameters are shown in parentheses. * n = programme-year combinations (calculated by summing the number of calendar years that each programme contributes data; a separate random effect is fitted for each programme-year combination). DTG = dolutegravir.

Figure S1 accompanies Figure 2 in the main text. This figure shows the absolute difference in viral suppression (in percentage points) associated with the switch from a lower threshold for viral suppression to the threshold of <1000 RNA copies/ml. The model predictions, for each validation data point, are compared against the actual increases. Conclusions are the same as for Figure 2 in the main text, although this figure shows more clearly the tendency for the models to ‘over-adjust’ in a number of the Latin American settings.

Figure S1: Percentage point changes in the proportion of adults who are virally suppressed when switching from a lower threshold to the <1000 threshold

Each panel compares the actual increase (‘validation data’) against the increase predicted by the adjustment model. In panels a and b, results are presented by country code (GT = Guatemala, HN = Honduras, NI = Nicaragua, VN = Vietnam, ZM = Zambia) and ART duration (in months). Confidence intervals around the validation data are not shown in panels c and d, as these estimates are based on large patient numbers and standard error estimates are <0.1%. VLS = viral load suppression.
